# A Proposed Link Between Acute Thymic Involution and Late Adverse Effects of Chemotherapy

**DOI:** 10.3389/fimmu.2022.933547

**Published:** 2022-07-01

**Authors:** Maria K. Lagou, Dimitra P. Anastasiadou, George S. Karagiannis

**Affiliations:** ^1^ Department of Microbiology and Immunology, Albert Einstein College of Medicine, Bronx, NY, United States; ^2^ Tumor Microenvironment and Metastasis Program, Albert Einstein Cancer Center, Bronx, NY, United States; ^3^ Cancer Dormancy and Tumor Microenvironment Institute, Albert Einstein Cancer Center, Bronx, NY, United States; ^4^ Gruss-Lipper Biophotonics Center, Albert Einstein College of Medicine, Bronx, NY, United States; ^5^ Integrated Imaging Program, Albert Einstein College of Medicine, Bronx, NY, United States

**Keywords:** chemotherapy, thymic involution, T cell, cancer immunoediting theory, immune surveillance, second primary malignacies

## Abstract

Epidemiologic data suggest that cancer survivors tend to develop a protuberant number of adverse late effects, including second primary malignancies (SPM), as a result of cytotoxic chemotherapy. Besides the genotoxic potential of these drugs that directly inflict mutational burden on genomic DNA, the precise mechanisms contributing to SPM development are poorly understood. Cancer is nowadays perceived as a complex process that goes beyond the concept of genetic disease and includes tumor cell interactions with complex stromal and immune cell microenvironments. The cancer immunoediting theory offers an explanation for the development of nascent neoplastic cells. Briefly, the theory suggests that newly emerging tumor cells are mostly eliminated by an effective tissue immunosurveillance, but certain tumor variants may occasionally escape innate and adaptive mechanisms of immunological destruction, entering an equilibrium phase, where immunologic tumor cell death “equals” new tumor cell birth. Subsequent microenvironmental pressures and accumulation of helpful mutations in certain variants may lead to escape from the equilibrium phase, and eventually cause an overt neoplasm. Cancer immunoediting functions as a dedicated sentinel under the auspice of a highly competent immune system. This perspective offers the fresh insight that chemotherapy-induced thymic involution, which is characterized by the extensive obliteration of the sensitive thymic epithelial cell (TEC) compartment, can cause long-term defects in thymopoiesis and in establishment of diverse T cell receptor repertoires and peripheral T cell pools of cancer survivors. Such delayed recovery of T cell adaptive immunity may result in prolonged hijacking of the cancer immunoediting mechanisms, and lead to development of persistent and mortal infections, inflammatory disorders, organ-specific autoimmunity lesions, and SPMs. Acknowledging that chemotherapy-induced thymic involution is a potential risk factor for the emergence of SPM demarcates new avenues for the rationalized development of pharmacologic interventions to promote thymic regeneration in patients receiving cytoreductive chemotherapies.

## Introduction

Despite the many advancements in the field of cancer therapeutics, including an array of targeted therapies and immunotherapies, chemotherapy still represents the frontier and standard-of-care therapeutic approach for the clinical management of the cancer patients (1). Today, a large number of cytoablative/cytostatic chemotherapies are available for clinical use in cancer patients, and are occasionally used alone, or more frequently under a combinatorial treatment strategy (2). These drugs are classified into five major classes (2), based on their mechanism of action: **(I)** Alkylating agents have the ability to covalently bind to and promote crosslinking of the two DNA strands *via* their alkyl group, thus leading to DNA strand break upon replication (i.e., during cell division) and triggering apoptosis (3). **(II)** Antimetabolites hinder the biosynthetic pathways of DNA/RNA, either because they inhibit enzymes that regulate DNA synthesis like DNA polymerase, or because they structurally resemble nucleobases/nucleosides lacking the proper chemical groups, thus preventing mitosis after their incorporation into the DNA (4). **(III)** Anti-microtubule agents interfere with microtubule dynamics, thus preventing key functions, such as the formation of the mitotic spindle during cell division and causing mitotic arrest (5). **(IV)** Topoisomerase inhibitors prevent the activity of topoisomerases, enzymes that physiologically introduce single- or double-strand breaks into the DNA to relieve strand tension and allow DNA to properly unwind during replication (6). **(V)** Cytotoxic antibiotics represent a large category of drugs with various modes of action, most notably prevention of cell division (7–9).

Beyond doubt, the survival rates of cancer patients have tremendously increased within the past decades due to more optimized and personalized use of chemotherapeutics, albeit with significant variations among different tumor types. Chemotherapy has even been successful in the radical treatment of certain tumor types, such as certain subtypes of testicular cancer and leukemias, although its therapeutic efficiency in most tumors of epithelial origin is rather limited, and at best suboptimal (10–15). An in-depth analysis of reasons behind the lack of its effectiveness is beyond the scope of this perspective. However, systemic toxicities rising from the lack of specificity in exclusively targeting neoplastic cells, drug resistance, and rapid drug metabolism/clearance of certain chemotherapeutics, signify only a few key reasons for their ineffectiveness against complete tumor eradication (2, 16, 17). More recent findings in preclinical mouse models of solid carcinomas suggest that chemotherapies may additionally promote neuroendocrine and stress responses, and elicit a proinflammatory cytokine surge, which together impede its short-term clinical benefits, by supporting a proangiogenic and prometastatic program in the tumor microenvironment, eventually leading to local and/or distant recurrence (18–21). Moreover, the long-term monitoring of cancer survivors (mostly pediatric cancer survivors) after years of receiving genotoxic treatments indicate a wide range of late adverse health effects, occurring mostly in, but not limited to, highly proliferating tissues, which include the hematopoietic, gastrointestinal, and reproductive systems. Such late adverse effects manifest as critical health issues in these patients, and include severe and long-term organ dysfunctions (including cardiotoxicity, neurotoxicity, nephrotoxicity, and hepatotoxicity, among others), infertility, cognitive impairment, and second primary malignancies (SPMs) (22–26). A thorough analysis on the occurrence and mechanisms behind all of the aforementioned adverse effects is beyond the scope of the current perspective. Here, we focus on the mechanistic origins of SPM, which represents one of the relatively understudied but most devastating late adverse effects in cancer survivors, as a paradigm for discussing the long-term consequences of chemotherapy on the immune system.

An SPM is defined as an unrelated primary cancer in a person who has experienced a different cancer sometime in their lifetime (22). By definition, SPM should be fundamentally distinguished from a secondary/metastatic cancer, especially if the latter occurs as a result of distant recurrence from a primary tumor, months or even years following treatment (27). The most prominent working model behind the development of such secondary cancers in the absence of a primary tumor relies on concrete, experimental evidence, collectively suggesting that cancer cell dissemination to distant metastatic sites, such as lungs, bone marrow, liver and brain, has occurred before the surgical excision or therapeutic management of the primary tumor (27). In this case, the long-term remission interval followed by relapse could be attributed to *cancer dormancy*, a stage of cancer progression, in which disseminated cancer cells either cease dividing (but survive in a quiescent state) or remain “locked” in a dynamic state, in which cancer cell proliferation balances cancer cell death (28, 29). Dormant cancers can remain clinically “silent” for months or even years, until the proper (micro)environmental conditions disrupt the dormancy program, and lead to a clinically overt tumor at the metastatic site (28, 29). On the contrary, SPM may rise on the same or a different organ and may either share a similar or different embryological origin with the first tumor; for example large B-cell lymphoma survivors are shown to be at high risk of developing colon, pancreas, breast (among other) tumors as late adverse SPMs (30). SPM is genetically distinct and independent from the first tumor that was experienced earlier in the patient’s life, and typically harbors mutations as a result of genotoxicity from the chemotherapies used for the treatment of the first tumor (31–33).

Nowadays, revolutionary treatments and improvement in patient care have allowed oncologists to face a constantly increasing long-lived population of cancer survivors. As such, the late adverse health effects of cytotoxic cancer treatments have become a recent clinical issue, due to the better clinical outcomes and favorable prognostic potential. Hence, there exists an unmet clinical need to unravel risk factors for such late adverse, and especially fatal, as in the case of SPMs, health effects. A consequent unmet clinical need would thus be to establish new prognostic biomarkers to stratify cancer survivors that are at high risk of developing such late adverse effects, with an ultimate vision of adapting their therapies, strengthening follow-up, and identifying novel pharmacological targets for medical interventions. The mission of the basic cancer scientist against this backdrop would thus be to provide a mechanistic insight on short- and long-term effects of cytotoxic cancer treatments on the immune system, as this appears to be the missing link for the development of devastating late adverse effects, such as SPMs. The current perspective offers a fresh working model, suggesting that acute thymic involution due to cytoreductive chemotherapy could significantly compromise the immune system of cancer survivors, thus leading to disturbed immune surveillance mechanisms and SPM development.

## Chemotherapy-Induced Second Primary Malignancy – Epidemiology

Although prominent risk factors for the development of SPMs include predisposing genetic factors and the patient’s lifestyle (as in the case of most primary cancers), it is important to mention that cytotoxic drugs that have been received for the clinical management of the first cancer and the patient’s age at the onset of such treatments, represent two of the most well-established, independent risk factors of SPM development (34–37). In the United States, cancer survivors have a 14% higher risk of developing SPM when compared to the general population (22). Interestingly, the cumulative risk to develop an SPM within 30 years following diagnosis of a primary pediatric malignancy is ~6.8% (22). Along the same lines, effective control of early-onset malignancies through radiation therapy and multiagent chemotherapy, has on one hand achieved significant increase in the 5-year survival of pediatric cancer patients (up to 80%), but has detrimentally increased the relative risk of developing SPM at 30 years after the diagnosis of the first tumor, by approximately 6-fold (38). Hence, cancer survivors receiving cytoreductive chemotherapy for the treatment of their primary cancer are at high risk of developing an SPM, even years after the completion of therapy.

Commonly observed SPMs following pediatric cancer treatment with alkylating agents are of hematologic origin and include among others acute lymphoid leukemia (ALL), acute myeloid leukemia (AML), chronic myelogenous leukemia (CML), and myelodysplastic syndrome (MDS) (39). Depending on the dose and/or possible combination with doxorubicin, alkylating agents may increase the risk of developing leukemias as SPMs by at least 5-fold (22). Other chemotherapeutics, such as cyclophosphamide can increase the risk of developing bladder cancer as SPM (22). The combination of alkylating agents with radiation therapy can also result in the manifestation of solid carcinomas as SPMs, including breast (40), lung (41), stomach (42), pancreas (43), thyroid (44), and colorectal cancer (45), as well as bone or other sarcomas (46, 47). For more details, the readers are encouraged to consult excellent reviews and surveys for the most common pediatric first and second primary malignancies in childhood cancer survivors, along with a thorough analysis of the risk factors associated with those (22, 38, 48). Interestingly, there is vigorous epidemiologic evidence suggesting that pediatric cancer survivors carry significant risk of mortality due to SPMs that present as adverse late health effects (22, 49–52). This brief epidemiological synopsis of SPM incidence, risk factors, and prognosis in cancer survivors is intended to merely provide the readers with a fundamental clinical basis, to better conceptualize the causative link between chemotherapy and emergence of late adverse effects (e.g., SPM development).

## Chemotherapy-Induced Second Primary Malignancy – Mechanistic Origins

As described in the landmark review by Hanahan and Weinberg (2011), cancer is now accepted as a multifaceted disease, organized by the acquisition of certain biological capabilities, broadly known as the “hallmarks of cancer”, and which can be summarized as the following: (i) Sustaining proliferative signaling, (ii) Evading growth suppressor mechanisms, (iii) Resisting cell death and apoptosis, (iv) Enabling replicative immortality, (v) Inducing angiogenesis, (vi) Activating invasion and metastasis, (vii) Reprogramming energy metabolism, and (viii) Evading immunological destruction (53). Underlying the acquisition of these acquired hallmark capabilities are two dimensions of tumor complexity. On one side is genome instability of transformed cells, which generates an essential genetic diversity (e.g., genomic mutations) that accelerates the acquisition of hallmark capabilities, while on the other side is a wide repertoire of recruited, seemingly normal cells that constitute the tumor microenvironment, and function as unwitting participants of cancer development and progression (53). Chemotherapy-based cancer treatments are highly genotoxic and are documented to increase the mutational burden of patients receiving them, thus providing an attractive rationale for the development of second independent malignancies as a late adverse effect. For instance, certain second primary leukemias developed by cancer survivors, including AML and MDS, present with deletion of 7q or monosomy 7 with normal chromosome 5, and deletions of 5q or monosomy 5, which are typical chromosomal aberrations due to prior exposure to alkylating agents (54). In another study, it was shown that topoisomerase II inhibitors, anthracyclines and mitoxantrone cause chromosomal translocations and chimeric rearrangements, leading to the manifestation of prolymphocytic leukemia as SPM (55–57). Topoisomerase II inhibitors have also been linked with translocations involving 11q23 or 21q22 in pediatric patients, leading to manifestation of AML within 1-5 years (58). Interestingly, genetic and epigenetic changes associated with cytotoxic treatments have also been reported for non-hematologic malignancies, such as pediatric ependymomas manifesting as SPMs, which depict hypermethylated phenotype leading to loss of tumor suppressor genes, such as CDKN2A, CDKN2B and p14ARF (59–61).

Although accumulation of such (epi)genetic defects due to chemotherapy treatment could partially explain early onset of SPMs, they cannot fully recapitulate the microenvironmental prerequisites that are essential for the development and progression of clinically overt tumors. Interestingly, the “immune surveillance” theory, originally proposed by Burnet and Thomas more than half a century ago, suggested that the immune system functions as a sentry in identifying and eradicating newly-emerging neoplastic cells. An extensive refining of this theory based on experimental observations, culminated into the foundation of the more concrete “cancer immunoediting” theory, consisting of 3 biologically distinct phases, to describe the many aspects of immune-tumor cell interactions (62–67). In the first phase, Elimination, newly risen neoplastic cells are eliminated by a competent immune system, collectively described as the “immune surveillance”. Intermittent tumor cells that manage to evade immunological destruction enter the second phase, Equilibrium, where immune-based elimination is balanced by the birth of new neoplastic cells. In the third phase, Escape, immunological “sculpting” allows tumors to progressively grow and lay the foundations for an immunosuppressive tumor microenvironment (62–67). As the primary site of T cell development and maturation, any intrinsic/extrinsic factors that negatively affect thymic integrity and functions could therefore affect the aforementioned immunoediting mechanisms, by shifting the balance toward the tumor-promoting end. Therefore, a critical question related to the origin of SPMs in cancer survivors is: “Could chemotherapy treatment have a long-lasting effect on the immune system, capable of hijacking the cancer immunoediting mechanism, thus facilitating SPM development in cancer survivors?”

To address this question, it is crucial to first recognize the key mediators of anticancer immunity. CD8^+^ T lymphocytes and natural killer (NK) cells encompass the backbone of anticancer immune responses and cancer immunoediting (62–64, 67–71). T cell-mediated responses in particular, are mediated by cytotoxic CD8^+^ T cells, which specifically recognize *via* their unique T cell receptor, one or more neoantigens on the cell surface of cancer cells (72–76). T cell-mediated anticancer immunity is supported by multiple stromal and immune cells, including cancer-associated endothelial cells and innate antigen-presenting cells (e.g., macrophages, dendritic cells), and leads to immunogenic cell death of tumor cells (77–79). To be able to recognize tumor cell neoantigens, a sufficient repertoire of T cell receptors and peripheral T cell pool with ability to monitor and elicit immunological attacks against neoplastic cells, must be generated in, and emerge from the thymus (80–82). As such, the thymus plays a critical role in the long-term establishment of anticancer immune surveillance and anticancer immunity (83). The thymus is a central lymphoid organ for T cell development, and signals derived from the thymic stromal epithelium are key determinants of thymocyte fate. The process of T cell development in the thymus is rather complex, and not the focus of the current perspective, but there are several checkpoints that determine efficient immune surveillance and anticancer immunity, such as: αβ-TCR gene rearrangement to acquire various specificities of neoantigen recognition, positive selection to achieve MHC restriction, and negative selection to establish central tolerance to self-antigens (84–87). Besides undergoing a natural decline termed age-related involution, the thymus is particularly sensitive to a variety of external stressors, as will be described in detail later, including cytoreductive chemotherapy, leading to its rapid involution and the consequent impairment of thymopoiesis (88–92).

Although thymic involution represents a logical mechanism for long-term immunosuppression and the failure of the immune system to control the emergence and survival of transformed cells, the link between thymus function and cancer development has been rather underrepresented in the “cancer immunology” literature. For instance, thymic involution could contribute to the long-term impairment of immune surveillance against tumor cells, enhanced ability of neoplastic cells to conceal their neoantigens and as such to evade immunological destruction, as well as the deployment of augmented immunosuppressive scaffolds in peripheral tissues (83). With regards to age-related thymic involution in particular, it has been documented that declined T cell-mediated immune surveillance is the outcome of reduced T cell repertoire diversity due to reduced thymic output, concurrent expansion of “immunosenescent” T cells expressing high levels of inhibitory checkpoint receptors (e.g., PD1), and a developmental shift towards immunosuppressive CD4^+^ T regulatory (Treg) cells, capable of suppressing CD8^+^ T cell functions in the periphery (83, 92–96). Therefore, the age-involuted thymus promotes the accumulation of multiple defects and the hijacking of the “cancer immunoediting machinery”, which together promote the development of clinically overt cancers. A critical question in the context of SPM development is: “Would chemotherapy-induced involution present similar defects in immune surveillance and the cancer immunoediting process, as seen in the case of age-related thymic involution?”

Although there is sufficient evidence of short-term consequences of chemotherapy on the immune system, less is known about how chemotherapy or other extrinsic stressors could affect the cancer immunoediting process, and as a consequence, the emergence of SPMs in cancer survivors. With regards to short-term consequences of chemotherapy on the immune system, detailed investigations have unraveled conflicting data. It has been suggested that chemotherapy can exert desirable immunological effects, by boosting tumor cell immunogenicity and promoting immunologic cell death (ICD) of tumor cells, which is characterized by the mobilization of innate immune responses and tumor-specific adaptive immune responses (97–99). For example, doxorubicin and cyclophosphamide are capable of causing the translocation of calreticulin, an endoplasmic reticulum chaperone, to the tumor cell surface, thus offering a signal for phagocytosis by dendritic cells and as a consequence, tumor antigen uptake and presentation (100, 101). Chemotherapy is also capable of increasing expression of MHC-I molecules on the tumor cell surface, thus turning them into attractive targets for cytotoxic CD8^+^ T cells, as well as of promoting the expression of NK stimulatory ligands, such as NKG2D, while suppressing NK inhibitory ligands (102–106). Finally, certain chemotherapeutics, including doxorubicin and cyclophosphamide, can enable type-I interferon signaling responses, and trigger macrophage recruitment, maturation, and NK cell proliferation (107, 108), thus establishing an immunostimulatory microenvironment. On the other side, chemotherapy has been documented to induce a systemic cytokine surge, and the subsequent recruitment of bone marrow progenitors, including proangiogenic/prometastatic TIE2^+^ monocytes, and myeloid-derived suppressor cells (MDSCs), which together promote a highly resilient and immunosuppressive tumor microenvironment (19, 21, 109–115). Moreover, certain chemotherapeutics, such as paclitaxel, can structurally mimic bacterial lipopolysaccharide, thus functioning as putative Toll-like receptor-4 (TLR4) agonists and leading to chronic inflammation, capable of hijacking the immune response against tumors (116–119). Besides the short-term effects, less has been unraveled on the immunological effects of chemotherapy over large periods of time.

However, indirect indications from epidemiologic data have hinted that cancer survivors may indeed suffer from suboptimal peripheral immune surveillance, due to receiving chemotherapeutics. For example, cancer survivors remain at elevated risk for developing infectious-related complications with a higher risk of persistent infections, and infection-related mortality, even years following chemotherapy (120–122), clearly suggesting that chemotherapy may exert long-term consequences to a patient’s immune system. In certain hematological malignancies, it has been shown that the type and dose of chemotherapy treatment can determine the rate and magnitude of lymphocyte recovery following treatment, and as such, the re-establishment of proper immune surveillance (123–125). These observations do not only suggest that early lymphocyte recovery may be a favorable prognostic indicator in these patients, but also highlight the importance of developing therapeutic strategies to support faster lymphocyte recovery to avoid early or late adverse effects of chemotherapy-compromised immune surveillance (123–125).

Valuable insights in this regard have been provided by many groups studying long-term consequences of thymic involution in peripheral immune surveillance. In general, thymic epithelial cells (TECs) are necessary for T cell differentiation and maturation, by providing key growth factors, chemokines, cytokines, and strictly regulated selection processes within the thymic environments. The phenotypic heterogeneity of cortical (cTEC) and medullary (mTEC) thymic epithelial cells is critical for the precision and coordination of intrathymic pathways leading to the development of mature T cells (80, 126–132). Several common immunosuppressants used to prevent allograft rejection such as cyclosporine, corticosteroids such as dexamethasone, and cytoreductive chemotherapies used for cancer treatment such as cyclophosphamide, are all known to cause impaired thymopoiesis and even autoimmunity, primarily by targeting cTEC and mTEC populations (133–138). Acute thymic involution as a result of cytoreductive chemotherapy leads to delayed recovery of T cells, with imminent consequences in the peripheral T cell pool and immune surveillance. In a non-pediatric setting, it has been demonstrated that repopulation of certain subsets of CD4^+^ T cells and B cells is delayed for almost a year following chemotherapy treatment in breast cancer patients (139). Although older studies have not looked into such extended periods of time, they have consistently reported that T cell recovery cannot be achieved between chemotherapy cycles, as opposed to the successful recovery of erythroid, myeloid and thrombocytic lineages (140, 141). In hematopoietic cell transplantation (HCT), cytoreductive chemotherapies are often used to prevent the transplant rejection, and as opposed to the fast recovery of non-lymphoid lineages post-chemotherapy, reconstitution of T cell adaptive immunity is profoundly delayed, often by a year or more (91, 142, 143). Post-chemotherapy T-cell deficiency in HCT recipients is not only associated with increased risk of infections and cancer relapse, but also with the development of SPMs, again due to failures in the cancer immunoediting mechanisms (141–147). Despite that all the non-T cell lineages are dependent on the bone marrow microenvironment for reconstitution following chemotherapy-mediated depletion, T lymphocytes are exclusively dependent on the thymus (148–150). The extensive delay in T cell reconstitution and the establishment of the peripheral T cell pool is therefore not attributed to impaired hematopoiesis, because the latter is restored soon after the termination of chemotherapy. Although the mentioned studies are quite indicative of the premise, the status of anticancer immune surveillance months or years following chemotherapy treatment has not been thoroughly assessed, and relevant animal models for such experimental testing are not, to our knowledge, standardized.

Besides impaired thymopoiesis leading to reduced peripheral T cell pool, chemotherapy-induced thymic involution may skew peripheral immune surveillance toward the development of precursor lesions for organ-specific autoimmune disease. As proof-of-concept, there is now clear epidemiologic evidence that post-chemotherapy rheumatism and other autoimmune syndromes may develop not only shortly, but even months or years, after completion of cytoablative treatments in (childhood) cancer survivors (151, 152). Mouse models of chemotherapy-induced thymic involution have determined that chemotherapy significantly obliterates the epithelial compartment of the thymus, most prominently the AIRE^+^ MHC-II^high^ mTEC subset, whose endogenous repair is a rather time-demanding process (91, 126, 153–156). An elegant study by Fletcher and colleagues (2009) has previously demonstrated that AIRE^+^ mTEC need approximately 7-10 days to be fully restored after treatment with immunosuppressive drugs or chemotherapeutics. Given that the restoration of AIRE^+^ mTEC is significantly delayed compared to the other TEC subsets, the authors concluded that a 7–10-day period of impaired or suboptimal AIRE^+^ mTEC function could be sufficient in allowing autoreactive T cells to escape the thymus and establish autoimmune lesions in the future (133). In general, the targeted deletion of the AIRE^+^ mTEC subset, or the targeting of the optimal expansion of AIRE^+^ mTEC *via* genetically engineered animals, both lead to organ-specific autoimmunity. For example, one study demonstrated that targeted deletion of the histone acetyltransferase KAT7 interferes with normal AIRE^+^ mTEC development in the thymic environment and causes profound lymphocyte infiltration into a variety of peripheral organs, such as the lung, liver, salivary glands, stomach, and lacrimal glands (157). Although an increased release of autoreactive T cells from the age-involuted thymus has been more strongly associated with chronic inflammation and autoimmunity (90, 158, 159), many investigators agree that the kinetically slow recovery of an acutely-involuted thymus can also provide a sufficient window to instigate the foundations of organ-specific autoimmunity.

**Figure 1:**
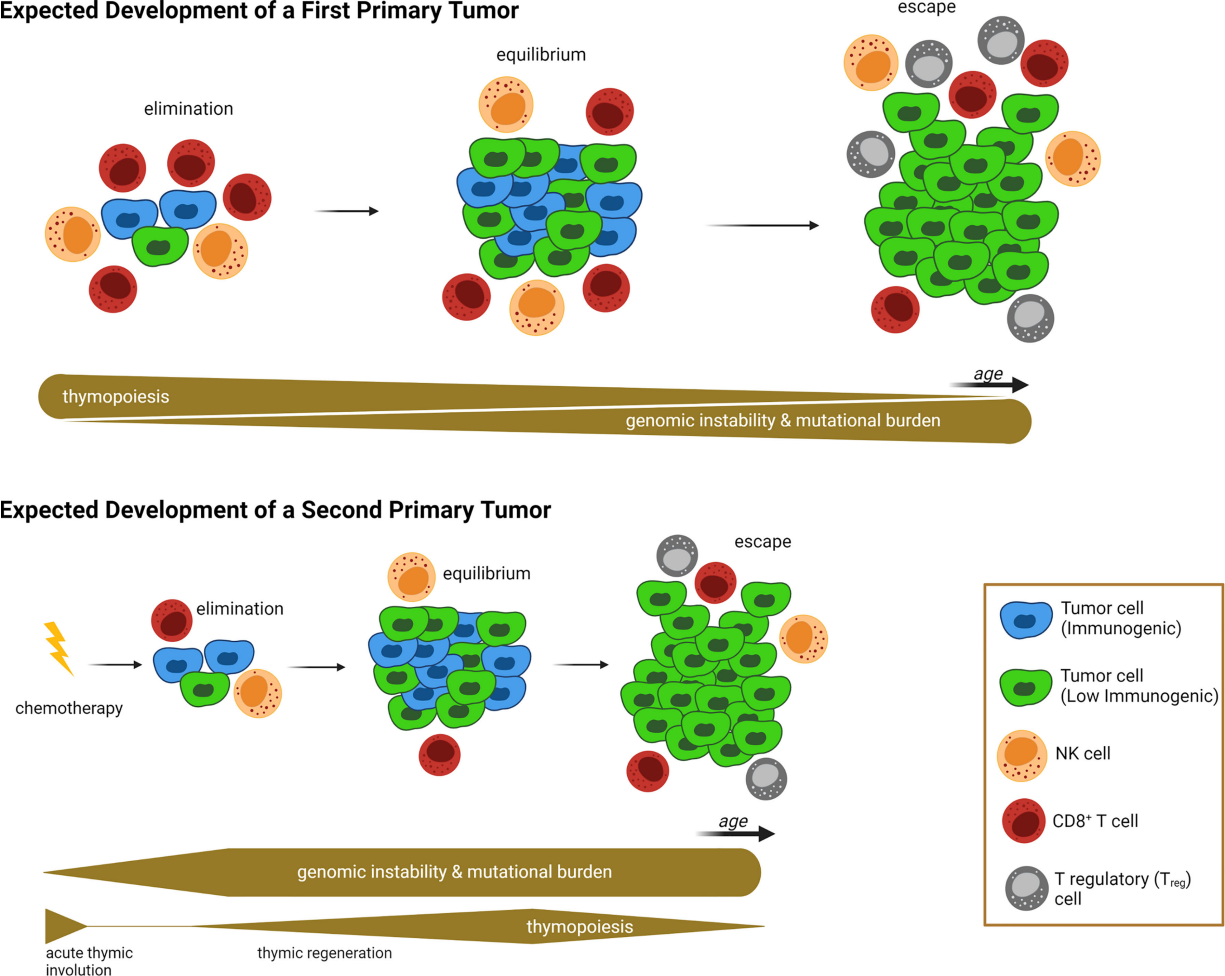
Proposed Link Between Acute Thymic Involution and Development of Second Primary Malignancy. In the absence of exposure to prior treatments with cytoablative chemotherapies due to a first-primary tumor (upper half of illustration), the emergence of nascent transformed cells is subjected to a “competent” cancer immunoediting process. At the beginning, the competent immune system can eliminate neoplastic cells *via* an efficient immune surveillance machinery. Then tumor cell growth is balanced by immunogenic cell death, described as equilibrium phase. And finally, immunosculpting leads to the escape phase, during which anticancer immunity fails to control tumor growth and creates a clinically overt tumor. The succession of these three phases is a long-lasting process with two main contributing factors: First, genomic instability is increased over time, leading to accumulation of driver mutations and genetic diversity that allows immunoevasive and immunosuppressive mechanisms to evolve (e.g. development of tumor cell clones with absent or low immunogenicity). At the same time, age-related thymic involution causes a decreased T cell peripheral pool and T cell receptor repertoires, leading to failure of immune surveillance and equilibrium mechanisms. In contrast, following exposure to a first-primary tumor and associated treatment with cytoreductive chemotherapy (lower half of illustration), the failure of the immune surveillance and equilibrium mechanisms occurs at a much earlier timepoint, allowing for the onset of clinically overt second primary malignancies (SPMs) at a younger age, compared to first-primary tumors (compare timelines between upper and lower half of illustration). Contributing factors for the SPM are the genotoxic nature of cytotoxic chemotherapy (which grants genomic instability and mutational burden at a very early onset), and chemotherapy-induced acute thymic involution causing impaired thymopoiesis, T cell receptor repertoires, and peripheral T cell pools, thus weakening immune surveillance mechanisms during elimination and equilibrium phases. *Relative thickness of gray bars underneath the timelines in each condition indicates the strength of thymopoiesis (upper bar), and genomic instability (lower bar) over time (not drawn to scale). Illustration designed with Biorender*.

*What lessons can be learned by studying thymic involution, either age-induced or chemotherapy-induced, in the context of impaired thymopoiesis and autoimmunity?* First, these studies collectively provide a proof-of-concept that damage/decline of various TEC components, including the most sensitive AIRE^+^ mTEC subset, could potentially manifest as prolonged “disturbance” of tissue immune surveillance, characterized by profound deficiencies in T cell receptor repertoire, peripheral T cell pool, and the presence of autoreactive cytotoxic CD8^+^ T cells. All these consequences can hinder the ability of the immune system to prevent nascent neoplastic cells *via* a competent immune surveillance, and to maintain control of tumor cell growth during the equilibrium phase of the cancer immunoediting process (154). Second, the thymus reaches its maximum relative size around birth and its maximum absolute size at puberty. As such, a significant impairment of thymopoiesis during this interval (i.e., the treatment of pediatric cancer patients with cytoreductive chemotherapy) would have a tremendous effect on the patient’s immune system. Because chemotherapy-compromised cancer immunoediting mechanisms may persist for a long period of time (e.g., years) in pediatric cancer survivors, neoplastic cells can escape elimination and equilibrium phases much earlier, thus manifesting as early onset SPMs or other adverse health effects (22, 120). This newly proposed working model of establishing a causative link between chemotherapy-induced thymic involution and SPM development, with the defective cancer immunoediting mechanisms serving as an intermediary, is illustrated in **Figure 1**.

## Chemotherapy-Induced Thymic Involution – Mechanistic Insights and Regeneration Strategies

**Figure 2:**
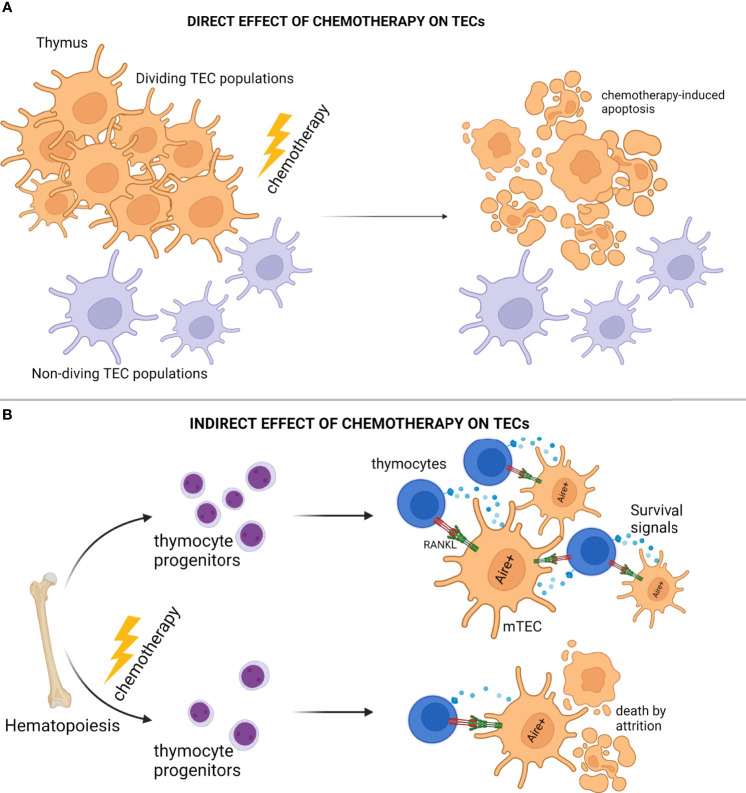
Modes of Thymic Epithelial Cell Death After Chemotherapy Treatment. **(A)** Cytoreductive chemotherapy non-specifically and unconditionally targets proliferation niches in the entire organism, and as such, insults TEC subsets in the act of cell division. **(B)** Cytoreductive chemotherapy suppresses bone marrow hematopoiesis and subsequent early thymocyte progenitor homing in the thymic microenvironment, thus disrupting thymocyte-derived prosurvival signals essential for TEC homeostasis, and causing “attritional” cell death to sensitive TEC subsets (e.g., AIRE^+^ mTEC). *Illustration designed with Biorender*.

The thymus is extremely sensitive to a wide array of external factors and stressors, including, but not limited to, acute/chronic infections, certain medications, glucocorticoids, cytoreductive chemotherapies, and even certain physiological states, such as pregnancy. Although these individual factors exert distinct effects on the thymic environment, they can all cause, in principle, extensive deterioration and/or complete elimination of the cTEC and mTEC compartments, leading to impaired thymopoiesis and escape of autoreactive T cells to the periphery (88, 91, 154, 155, 160, 161). In the case of cytoreductive treatments, the initial effect is dependent on the chemotherapy's mechanism of function, which is typically disruption of one or more steps associated with cell division, and as such the proliferating thymic epithelial cell pool is directly assaulted shortly after administration (88, 91, 161, 162). In general, chemotherapies that function by perturbing cell division will systemically suppress most of the actively proliferating niches, including the hematopoietic niche, which often leads to impaired multi-lineage hematopoiesis (163–165). Impaired lymphopoiesis leads to diminished mobilization of lymphocyte progenitors, and as such, it also leads to reduced homing of early thymic progenitors (ETPs) in the thymus environment (166, 167). Hence, the devastating effects on thymic architecture and function observed during chemotherapy are primarily related to its direct mechanism of action on proliferating niches in the mammalian body, and manifest as acute reduction of both thymocytes and TECs **(** **Figure 2A** **)**.

Several investigations have interestingly revealed that when compared to cTEC subsets, AIRE^+^ mTEC are more sensitive to stressor-mediated destruction, a feature that typically manifests as disproportional reconstitution of corticomedullary ratio with detrimental, long-term, organ-specific repercussions, such as development of autoimmunity and leukemic transformation (156, 162, 168, 169). Underlying this biased inefficiency of mTEC to repair from acute thymic involution may be indirect consequences of cytoablative treatments. Although, chemotherapies lead to severe reduction of thymocytes in the thymic environments as mentioned above, it is now well known that thymocytes and TECs participate in reciprocal signaling loops providing trophic and survival factors to one another (129, 131). For example, AIRE^+^ mTEC are strongly dependent on RANK ligands (RANKL) provided by the single positive CD4^+^ thymocytes and type 3 innate lymphoid cells (ILC3) for proliferation/differentiation and TEC regeneration (131, 170, 171). Therefore, chemotherapy-mediated disruption of lymphopoiesis will result in the elimination of lymphocyte homing and as such, the elimination of the TEC survival signals. In conclusion, besides the well-reported and direct mechanisms for chemotherapy-induced immunotoxicity, cytoablative treatments may also lead to prolonged “attritional” death of mTEC subsets due to the selective elimination of essential microenvironmental factors, such as RANKL **(** **Figure 2B** **)**.

Naturally, the thymus has the endogenous capacity to regenerate from the loss of thymic epithelium (91, 172–174), although the time interval necessary for the completion of endogenous repair might be sufficient to cause critical failures in the aforementioned cancer immunoediting mechanisms, as already mentioned in the previous chapter. A recent, but active area of research, relies on the development of pharmacological interventions to facilitate thymic regeneration following chemotherapeutic or other cytotoxic insults. From the viewpoint of the current perspective, such strategies would be rather beneficial by boosting thymic functions and enhancing peripheral immune surveillance mechanisms in cancer survivors, to prevent early onset of SPMs and other late adverse effects of chemotherapy. In the following paragraphs, we briefly discuss the underlying principles of well-established regeneration strategies following acute thymic involution.

**Figure 3:**
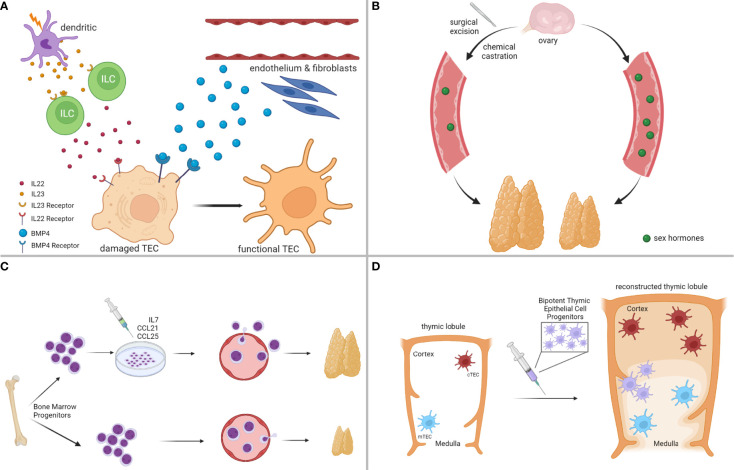
Strategies for Enhancing Thymus Regeneration Following Chemotherapy. **(A)** Examples of thymus regeneration strategies targeting thymic stromal cell networks activated in endogenous thymic repair. **(B)** Examples of thymus regeneration strategies targeting negative feedback loops on thymus size/function from sex hormones. **(C)** Examples of thymus regeneration strategies involving the transplantation of (pre-conditioned) bone marrow-derived thymocyte progenitors. **(D)** Examples of thymus regeneration strategies that are not dependent on the endogenous thymus, such as transplantation of bipotent thymic epithelial cell progenitors to reconstitute thymus lobules and functions. *Illustration designed with Biorender*.

A significant number of regeneration strategies has focused on targeting cells, essential for thymic architecture and function, most notably cTEC and mTEC subsets (91), as thematically illustrated in **Figure 3A**. For instance, favorable outcomes have been reported from exposure to Fibroblast Growth Factor-7 (FGF7) (175, 176), Insulin-like Growth Factor-1 (IGF1) (177), Wingless-related Integration site-4 (WNT4) (178), Bone Morphogenetic Protein-4 (BMP4) (174), and Interleukin-22 (IL22) (172). Most of these endogenous pathways orchestrate complex intrathymic circuitries, simultaneously involving multiple stromal epithelial, stromal non-epithelial (e.g., endothelial cells, mesenchymal cells), and immune cells (e.g., macrophages, dendritic cells, innate lymphoid cells), which cooperate to support the reconstitution of the appropriate thymic infrastructure for T cell development. For example, following radiation-induced thymic involution, a subset of dendritic cells secretes interleukin-23 (IL23) in the thymic environment, which stimulates innate lymphoid cells to subsequently secrete IL22, in turn promoting the survival and proliferation of radiation-affected TECs (172, 179). In another study, BMP4 was shown to be primarily secreted by the intrathymic endothelium and mesenchymal fibroblasts, and was significantly overexpressed following acute thymic involution to support the replenishment of BMPR2^high^ cTEC populations, eventually facilitating thymic repair (174). A thorough analysis of all implicated studies in this category is beyond the scope of this perspective, but suffice is to claim that a concrete understanding of the paracrine/juxtacrine intrathymic milieu is paramount for the successful design of therapeutic modalities.

Because thymus physiology is under constant neuroendocrine control (180), a separate class of regeneration strategies has proposed the development of hormonal therapies to systemically control thymus growth (91), as thematically summarized in **Figure 3B**. Sex steroids have a negative impact on thymus function, and experimental models of chemical or surgical ablation of sex steroids have given positive results in thymic regeneration following acute thymic involution (153, 181–183). However, despite the beneficial effects of sex steroid inhibition in lymphoid potential and hematopoietic stem cell function, more studies need to be conducted in this direction, because animal models of castration often lead to increased release of autoreactive T cells (91, 155, 184). These findings raise the concern that regeneration strategies should carefully balance lymphocyte progenitor supply with the size of the thymic epithelial compartment to avoid detrimental consequences, such as autoimmunity.

Less explored thymic regeneration strategies include chemokine and cytokine therapy, to improve homing of bone marrow lymphocyte progenitors and expansion of thymic T cell precursors in the thymus (91), as exemplified in **Figure 3C**. The mechanistic principles behind the elicitation of such strategies rely on the fact that chemotherapy has a detrimental effect on bone marrow hematopoiesis, and the restoration of lymphopoiesis is rather restricted following the termination of the cytotoxic result (185, 186). A prominent example of such an approach includes pretreatment of bone marrow progenitors with CCL25 and CCL21 before autologous transplantation, to rescue their homing capacity in the thymus after exposure to the cytoreductive insult (187). Another strategy that circumvents hematopoietic cell transplantation involves the administration of IL7, a cytokine, endogenously secreted by cTEC subsets to promote T cell proliferation and expansion, innate lymphoid cell development, and lymphoid tissue organization (188–190).

Other even less explored, but emerging strategies involve the development of artificial thymic niches to circumvent the reliance on the endogenous thymus upon cytoreductive insult (191), and the transplantation of bipotent TEC progenitor (TECP) cells to reconstruct the entire thymic environment (192), both of which show great promise **(** **Figure 3D** **)**. Taken together, our goal in this section was not to provide an exhaustive discussion of all available regenerative strategies that are currently explored to boost thymic function following cytotoxic insults. Instead, we hoped to offer a brief overview of the most promising pharmacologic interventions that could help restore the cancer immunoediting mechanisms in cancer survivors receiving chemotherapy.

## Criticisms of the Proposed Model and Future Repercussions

The cancer immunoediting process functions as a devoted sentinel under the auspices of a highly competent immune system to put a tissue barrier on tumor development and progression. In this hypothesis and theory article, we explored the premise that cancer survivors who have received cytoreductive chemotherapy may present with multiple defects on the cancer immunoediting mechanisms, as a result of chemotherapy-induced thymic involution. These observations would further imply that the onset of late adverse effects of chemotherapy is not exclusively attributed to the genotoxic potential of these drugs, but also to their negative impact on thymic functions and T cell development. At this point, our proposed model is not intended to be a comprehensive and exhaustive analysis of all genomic and contextual intricacies governing the defects of the cancer immunoediting process that could lead to SPMs after chemotherapy. Rather, we have laid the groundwork for future expansions of the proposed model. For instance, we focused primarily on CD8^+^ T cell-mediated anticancer immunity and immune surveillance to discuss the relevant defects on the cancer immunoediting mechanisms. However, there is now compelling evidence that both NK cells and NKT cells comprise a substantial component of the anticancer immune response, and cancer immune surveillance mechanisms (193–197), suggesting that the effects of chemotherapy on conventional intrathymic pathways for T cell development could be only one side of the coin. As such, it would be important that future investigations focus on systematic immunology studies to address the impact of chemotherapy on the immune system.

The proposed model primarily focuses on the impact of chemotherapy on cancer immunoediting mechanisms, from the viewpoint of prolonged impaired thymopoiesis after chemotherapy. Our model, however, did not discuss the impact of chemotherapy on the quality of thymopoiesis upon chemotherapy treatment. A large body of evidence now suggests that age-related involution is related to immunosenescence, which is translated not only in defects on numbers of peripheral T cells during involution, but also in increased numbers of T regulatory cells and markers of T cell exhaustion in the periphery (83, 94, 198–204). In our opinion, “immunosenescence” has not been adequately addressed in the context of acute thymic involution, but regardless, it should be taken into account during the experimental design of future thymus regeneration strategies.

Our proposed model has not made clear distinctions between types or schemes of chemotherapy and specific defects on cancer immunoediting mechanisms and development of SPMs. In part, this is due to the fact that not many such studies currently exist. However, it would be an oversimplification to claim that all chemotherapies exert similar effects or have the same capacity to inflict SPMs, given that, for example, there are well-known specific mutations tied to specific drug classes (22). In addition, chemotherapies may potentially affect thymic environments in a heterogeneous manner. As mentioned, paclitaxel has been shown to function as a lipopolysaccharide mimetic, thus promoting an acute proinflammatory milieu by functioning directly as a TLR4 agonist, besides the traditional mechanism of microtubule stabilization (116–119). Many groups have compared neoadjuvant versus adjuvant chemotherapy settings, either on the local tumor microenvironment or the systemic tumor “macroenvironment”, and also reported fundamental epidemiological differences in their response (19, 115, 205–212). Because the choice of cytoreductive treatments could have a unique effect on thymopoiesis, such considerations should be carefully taken into account as the scientific community moves forward in the field, to properly enrich and revisit our currently proposed model.

Our proposed model focuses on pediatric cancer patient survivors, to propose a causative link between acute thymic involution and defective cancer immunoediting mechanisms leading to SPMs. Nevertheless, SPM development also occurs in non-pediatric patients, and similar mechanisms could also be relevant in these populations (41, 213–221). The pediatric cancer survivor paradigm was easier to discuss in our model, first because there are long-term follow-up epidemiological data that can be used as a better proof-of-concept (222–224), and second, because thymic functions are relatively stronger in childhood, as compared to other ages (201). However, due to scientific advancements, oncologists are nowadays faced with an increasing population of cancer survivors at all ages, and as such, we anticipate that studies on acute thymic involution will eventually become relevant for older cancer survivors.

To conclude, acknowledging that chemotherapy-induced thymic involution is a risk factor for the emergence of SPMs opens a new avenue for the rationalized development of pharmacologic interventions to promote thymic regeneration in patients receiving cytoreductive chemotherapies. Here, we articulated that this research field is promising and exciting, and we further anticipate that it will be at the frontier of personalized medicine in the next decade.

## Data Availability Statement

The original contributions presented in the study are included in the article/supplementary material. Further inquiries can be directed to the corresponding author.

## Author Contributions

GK conceptualized the working hypothesis/model. ML designed all illustrations; ML, DA, and GK wrote, drafted, and edited the manuscript. All authors contributed to the article and approved the submitted version.

## Funding

This work was supported by a new investigator start-up fund (PI: GK) from the Albert Einstein Cancer Center (AECC) grant number: NIH NCI P30 CA013330-49.

## Conflict of Interest

The authors declare that the research was conducted in the absence of any commercial or financial relationships that could be construed as a potential conflict of interest.

## Publisher’s Note

All claims expressed in this article are solely those of the authors and do not necessarily represent those of their affiliated organizations, or those of the publisher, the editors and the reviewers. Any product that may be evaluated in this article, or claim that may be made by its manufacturer, is not guaranteed or endorsed by the publisher.
